# METTL3 potentiates resistance to cisplatin through m^6^A modification of TFAP2C in seminoma

**DOI:** 10.1111/jcmm.15738

**Published:** 2020-08-28

**Authors:** Jingchao Wei, Yinghao Yin, Jun Zhou, Hanfei Chen, Jingxuan Peng, Jianfu Yang, Yuxin Tang

**Affiliations:** ^1^ Department of Urology The Second Affiliated Hospital of Zhejiang University School of Medicine Hangzhou China; ^2^ Department of Urology The Third Xiangya Hospital of Central South University Changsha China; ^3^ Department of Urology The Fifth Affiliated Hospital of Sun Yat‐sen University Zhuhai China; ^4^ Guangdong Provincial Key Laboratory of Biomedical Imaging The Fifth Affiliated Hospital of Sun Yat‐sen University Zhuhai China

**Keywords:** chemoresistance, m^6^A, METTL3, seminoma, TFAP2C

## Abstract

Testicular germ cell tumours (TGCTs) rank as the most common malignancy in men aged 20‐34 years, and seminomas are the most type of TGCTs. As a crucial anti‐tumour agent with explicit toxicity, cisplatin may render resistance through intertwined mechanisms, even in disease entities with high curative ratio, such as seminoma. Previously, we established cisplatin‐resistant seminoma TCam‐2 (TCam‐2/CDDP) cells and showed that epigenetic regulations, such as non‐coding RNA (ncRNA) interactions, might orchestrate cell fate decisions in the cisplatin treatment context in seminoma. N6‐methyladenosine (m6A) is the most prevalent internal modification in mRNA. In the present study, we assessed cisplatin resistance in seminoma from the perspective of m^6^A, another manner of epigenetic modification. The global m^6^A enrichment of TCam‐2 and TCam‐2/CDDP was depicted. Then, we elucidated whether transcription factor‐activating enhancer‐binding protein 2C (TFAP2C) was functionally m^6^A‐modified by methyltransferase‐like protein 3 (METTL3), which acted as an m^6^A ‘writer’, and insulin‐like growth factor 2 mRNA‐binding protein 1 (IGF2BP1), which acted as an m^6^A ‘reader’. Enhanced stability of TFAP2C mRNA promoted seminoma cell survival under cisplatin treatment burden probably through up‐regulation of DNA repair‐related genes. Hopefully, this study will help improve our understanding of the subtleties of the tumour cellular coping strategy in response to chemotherapy. Targeting factors that are involved in m^6^A methylation may be an effective strategy for circumventing cisplatin resistance in seminoma.

## 1 INTRODUCTION

Testicular germ cell tumours (TGCTs) are the most common malignancy and the leading cause of death from solid tumours in young men.^1^, ^2^ These tumours peak during the third decade of life, and an increasing incidence has been reported widely.^3^ Histologically, TGCTs are subdivided into various elements. Type II TGCT refers to seminomas and non‐seminomas of (predominantly) adolescents and adults. This type of TGCT has gained increasing attention in recent years, and more and more studies led to significant increases in understanding of Type II TGCT. Among the subtypes, seminoma makes up for nearly half of all TGCTs.^4^ Seminomas generally arise in the fourth decade of life and are averagely diagnosed in patients a decade older than those with non‐seminomas. It is also known to be bilateral in 3% of cases and classically manifests as a painless, palpable, solid mass. Seminomas uncommonly present with symptoms related to metastatic disease. Most patients are evaluated with physical examination and scrotal ultrasonography. Pure testicular seminomas do not have specific serum tumour markers, but in certain cases can produce a small amount of βHCG (β‐subunit of human chorionic gonadotropin). Primary treatment for seminoma includes surveillance, radiotherapy and chemotherapy. Since its discovery, cisplatin (cis‐diamminedichloroplatinum II, CDDP) has been one of the most widely used chemotherapy agents in multiple types of tumours, including seminoma.^5^ The majority of seminoma patients treated with platinum‐based therapy achieved a favourable response.^6^, ^7^ However, like a two‐edged sword, CDDP has dual roles in anti‐tumour therapy. The main problems concurrent with CDDP utilization are toxicity and resistance. CDDP failed in some patients after the initial treatment.^8^ Late relapse, which is defined as tumour recurrence more than 2 years after complete remission following primary treatment including chemotherapy, occurred in some cases, usually accompanied by chemoresistance,^9^ and patients with refractory tumours showed a poor prognosis.^1^ In patients whose seminoma progresses during or immediately after cisplatin‐based chemotherapy, the outlook is dismal. Refractoriness to cisplatin‐based chemotherapy is the most important factor associated with the poor outcomes. Currently, no molecular aberrations that could be used for targeted therapy have been identified and studies of the use of drugs including cisplatin have not been successful. Circumventing CDDP resistance remains a goal for seminoma chemotherapy.

Generally, drug resistance is attributed to many mechanisms, including cell heterogeneity, genetic alteration and epigenetic modification.^10^, ^11^ In TGCTs, resistance to CDDP may result from decreased drug uptake, enhanced metabolic turnover, diminished apoptosis and potentiated DNA repair.^12^, ^13^ Previously, we showed that sensitivity of seminoma to CDDP is regulated by testis development‐related gene 1 (TDRG1), which acts as an oncogene in testis.^14^, ^15^, ^16^ Furthermore, this effect of TDRG1 on CDDP chemosensitivity has been found to be regulated by lncRNA H19 and miRNA‐106b‐5p.^15^ Of note, tumour cells are often versatile in response to host microenvironment changes, such as high treatment burden.^17^ We believe that elucidating the mechanisms potentiating seminoma resistance to CDDP from another point of view will improve our understanding regarding how tumour cells respond to CDDP treatment.

N6‐methyladenosine (m^6^A) is a specific RNA modification manner present prevalently, which is observed in numerous eukaryote species, even viruses.^18^, ^19^, ^20^, ^21^ The presence of m^6^A methylation of RNAs has been recognized for more than 40 years.^22^, ^23^, ^24^ m^6^A methylation mostly occurs at the consensus motif of RRm6ACH ([G/A/U][G/A]m6AC[U/A/C]), and it significantly clusters around the stop codon and 3′ untranslated region (3′UTR). M6A methylation is dynamic and reversible in mammalian cells, and it has been considered as another form of epigenetic regulation. ‘writers’, ‘erasers’ and ‘readers’ of m6A methylation are proteins that can add, remove or recognize m6A‐modified sites and alter biological processes accordingly. m^6^A methylation has been shown to function throughout the whole RNA life cycle, including RNA splicing, nuclear transportation, RNA stability and translation.^25^, ^26^, ^27^ Deregulation of m6A modification has been implicated in many diseases including cancers. Nettersheim et al have analysed distribution of m6A in TGCT entities.^28^ However, the role of m^6^A in seminoma is not well understood although evidence shows that abundance of m^6^A was higher in seminoma compared to non‐seminoma subtypes of TGCTs, indicating that m^6^A might contribute to seminoma phenotype maintenance.^29^, ^30^ Given the research progress on m^6^A in regulating multiple biological processes, it is reasonable to speculate that m^6^A might affect cellular response to chemotherapy in seminoma.

In this study, in an attempt to better understand the mechanism of chemoresistance in seminoma, we compared the m^6^A enrichment between TCam‐2 and the previously established CDDP‐resistant TCam‐2 (TCam‐2/CDDP) cell lines and explored the underlying roles of m^6^A in chemoresistance of TCam‐2/CDDP cells. Two molecules, methyltransferase‐like protein 3 (METTL3) and insulin‐like growth factor 2 mRNA‐binding protein 1 (IGF2BP1), were found to play crucial roles in potentiating resistance to CDDP through m^6^A methylation of transcription factor‐activating enhancer‐binding protein 2C (TFAP2C) in seminoma. Besides, we found that two DNA repair‐related genes were up‐regulated in the CDDP treatment context and were correlated with m^6^A methylation of TFAP2C, indicating the contribution of the DNA repair mechanism in cellular resistance to CDDP in seminoma.

## 2 MATERIALS AND METHODS

### 2.1 statement

The Institutional Research Ethics Committee of The Fifth Affiliated Hospital of Sun Yat‐sen University approved the study (no. 2019‐L020‐1). All experiments adhered to the principles set forth in the Declaration of Helsinki.

### 2.2 Cell culture

The human TCam‐2 cell line was kindly gifted by Dr Riko Kitazawa. Cell culture conditions and the establishment of the TCam‐2/CDDP cell line were described previously.^15^ Briefly, cells were maintained in complete medium (Roswell Park Memorial Institute Medium‐1640 with 10% foetal calf serum) with 5% CO_2_ at 37°C.

### 2.3 Plasmid and siRNA

For construction of the METTL3 overexpression plasmid, please refer to Supporting Doc 1. The nucleotide sequences of the siRNAs (GenePharma, Shanghai, China) were as follows: METTL3 siRNA: sense 5′‐GCAAGUAUGUUCACUAUGATT‐3′; IGF2BP1 siRNA: sense 5′‐UGGAUGCUACGAGUAUAAATT‐3′; TFAP2C siRNA: sense 5′‐UCAGCUCUACGUCUAAAUATT‐3′; Negative control siRNA: sense 5′‐UUCUCCGAACGUGUCACGUTT‐3′. Cells showing logarithmic growth were seeded into six‐well plates, at 5 × 10^4^ cells per well, cultured for 24 hours and then transfected with 5 μL Lipofectamine 2000 (Invitrogen, Carlsbad, CA, USA) for siRNA 100 pmol or 2.5 μg plasmid according to the manufacturer's instructions.

### 2.4 **Quantitative real**‐**time PCR (qPCR), immunoblotting and immunohistochemistry**

The procedures for these assays can be found elsewhere as we have previously reported them.^15^, ^31^ qPCR assays were carried out using three technical replicates and three independent biological replicates. Total RNA was extracted using the TRIzol reagent.

mRNA was reverse‐transcribed with 1 μg of total RNA by using the FastQuant RT Kit with gDNase (Tiangen, Beijing, China). RT‐qPCR was performed with SuperReal PreMix Plus (Tiangen, Beijing, China) using a LightCycler 96 thermocycler (Roche, Basel, Switzerland). The nucleotide sequences of the primers were as follows: TFAP2C‐F: 5‐TCAGTCCCTGGAAGATTGTCG‐3, TFAP2C‐R: 5‐CCAGTAACGAGGCATTTAAGCA‐3; GAPDH‐F:5‐GGAGCGAGATCCCTCCAAAAT‐3, GAPDH‐R:5‐GGCTGTTGTCATACTTCTCATGG‐3. Annealing temp: TFAP2C 60.9°C; GAPDH 61.0°C. Cycles: 40. The relative gene expression levels were normalized to the housekeeping gene GAPDH and calculated by using the 2^−ΔΔCt^ method. For the immunoblotting assay, GAPDH protein was used as a loading control. The information of the primary antibodies was as follows: anti‐METTL3 (1:500; Proteintech Group, Rosemont, IL, USA), anti‐TFAP2C (1:1000; Abcam, Cambridge, UK), anti‐GAPDH (1:4000; Abcam), anti‐IGF2BP1 (1:500; Santa Cruz Biotechnology, Dallas, TX, USA), anti‐WEE1 (1:500; Abcam) and anti‐BRCA1 (1:2000; Abcam). Image J software was used for analysation. In the immunohistochemistry (IHC) assay, the information of the primary antibodies was as follows: anti‐Ki‐67 antibody, 1:100 dilution and GeneTex (Irvine, CA, USA). Tissue was fixed overnight in 4% paraformaldehyde (PFA) and embedded in paraffin. Paraffin sections were cut at a 4 μm thickness. Then, tissue was deparaffinized and rehydrated with ethanol. Antigen retrieval was performed by placing the chips in 0.01 M citrate buffer (pH 6.0) before microwave heating for 20 minutes. Normal goat serum (10%) was utilized for 30 minutes to block non‐specific binding sites. Subsequently, the samples were incubated with primary antibodies at 4°C overnight. The primary antibodies were visualized by adding a secondary biotin‐conjugated antibody followed by an avidin/biotin/peroxidase complex (Vectastain ABC Elite kit; Vector Laboratories lnc, Burlingame, CA, USA) and substrate (Vector NovaRED, Vectastain). Image J software was used for analysation.

### 2.5 Cell viability assay

To evaluate the effects of CDDP on cell viability, the CCK‐8 assay was performed. TCam‐2/CDDP cells were seeded into 96‐well culture plates at a density of 5000 cells/well and cultured for 24 hours. Then, cells were cotreated with CDDP in 1640 medium for 0, 12, 24, 48 and 72 hours. Thereafter, 10 μL of CCK‐8 (Dojindo Laboratories, Kumamoto, Japan) solution was added to each well and mixtures were incubated at 37°C for additional 1 hour. The optical density (OD) at 450 nm was read with a microplate reader (Bio‐Tek ELX‐800, Winooski, VT, USA).

### 2.6 Flow cytometry assay (FCM, cell apoptosis analysis)

Briefly,^15^ after drug treatment and incubation for 72 hours, cells were collected and then stained with Annexin V‐FITC (fluorescein isothiocyanate)/PI (Sigma‐Aldrich, St. Louis, MO, USA) staining for apoptosis analysis by FACSCalibur flow cytometry (BD Biosciences, San Jose, CA, USA).

#### 2.6.1 Gamma‐H2AX immunofluorescence assay

Samples were fixed overnight in 4% paraformaldehyde and embedded in paraffin.

Paraffin sections were prepared at a 4 μm thickness. After heat‐mediated antigen retrieval with citrate buffer (pH 6.0) was conducted, the sections were incubated with overnight at 4°C with the anti‐human gamma‐H2AX (1:50; Abcam) primary antibody. The secondary antibody Alexa Fluor^®^ 488 Goat Anti‐Rabbit IgG was used for 1 hour in the dark. Then, DAPI (1:1000; Abcam) was added and the samples were incubated for 10 minutes in the dark. Immunofluorescence staining was analysed on an inverted fluorescence microscope (Leica, Wetzlar, Germany). Image J software was used to count the positive cells.

### 2.7 Measurement of total m^6^A and m^6^A^+^ TFAP2C mRNA levels

Total m^6^A content was measured in 200 ng aliquots of total RNA extracted from TCam‐2 and TCam‐2/CDDP using an m^6^A RNA methylation quantification kit (Epigentek, NY, USA) according to the manufacturer's instructions. To measure the m^6^A^+^ TFAP2C mRNA levels, m^6^A immunoprecipitation was performed (see below). A 1‐µg aliquot of the m^6^A antibody was conjugated to protein A‐agarose slurry overnight at 4°C. A 100 μg aliquot of total RNA was incubated with the antibody in immunoprecipitation buffer supplemented with RNase inhibitor for 3 hours at 4°C. RNA was eluted from the beads by incubation in 300 μL of elution with 4.2 μL of proteinase K for 1.5 hours at 50°C, and m^6^A^+^ RNA was purified by phenol/chloroform extraction and analysed by qPCR. For primers, annealing temp and cycles, see above.

### 2.8 RNA‐binding protein immunoprecipitation assay

RNA‐binding protein immunoprecipitation (RIP) assay was performed using the EZ‐Magna RIP™ RNA‐Binding Protein Immunoprecipitation Kit (Millipore, Billerica, MA, USA) according to the manufacturer's instructions, and the procedure was described previously.^15^

### 2.9 RNA stability assay

Cells were treated with 5 μg/mL actinomycin D to inhibit transcription. TCam‐2/CDDP cells were lysed after 0, 2, 4, 6, 8 and 12 hours following actinomycin D treatment. RNA was isolated and reverse‐transcribed as previously described. Transcript levels were determined using qPCR and normalized to levels of 18s RNA. Fold change was calculated relative to TFAP2C levels at the 0 hour time‐point.

### 2.10 Chromatin immunoprecipitation

Chromatin immunoprecipitation (ChIP) assay was performed using the Pierce Agarose ChIP Kit (Thermo Scientific, Rockford, IL, USA). Briefly, cells were treated with METTL3 overexpression plasmids and METTL3 overexpression plasmids were combined with IGF2BP1 siRNAs or control vehicles for 48 hours and then fixed with 1% formaldehyde to cross‐link DNA and protein. The chromatin was digested and 10% of the chromatin fragments were used as input DNA. Immunoprecipitation was performed with either an anti‐TFAP2C antibody or an immunoglobulin G control (Cell Signaling Technology, Beverly, MA, USA). The immunoprecipitated DNA was then quantitated using qPCR with specific primers for the WEE1 promoter (F:5′‐ATCGCGTAGCTGGTCCTTC‐3′; R: 5′‐TCCTCAGGTCCAGTCTCAGG‐3′), BRCA1 promoter (F: 5′‐GCTGGCTTCACCTAGTGGAT‐3′; R: 5′‐ACCCCGCTTGAA TTCTCAC‐3′). Annealing temp: WEE1 60.4°C; BRCA1 59.7°C. Cycles: 40. The enrichment of targeted genomic regions was normalized with input DNA and presented as a value relative to the immunoglobulin G control.

### 2.11 Xenografts

The Department of Laboratory Animals, Central South University, provided a total of 24 male BALB/c nude mice. The in vivo experiments were conducted in compliance with the recommendations in the Guide for the Care and Use of Laboratory Animals of CSU. Male mice were subcutaneously injected with tumour cells near the limbs to establish xenografts (1 × 10^6^/mouse, 0.2 mL for each injection site; METTL3‐overexpressing TCam‐2/CDDP cells were inoculated once at the initial time and IGF2BP1‐inhibited TCam‐2/CDDP cells were inoculated every 3 days). For CDDP treatment, mice were intraperitoneally injected with three doses of CDDP (6 mg/kg bodyweight) or vehicle every week after tumour cell inoculation. Mice were killed 25 days after tumour cell inoculation, and tumour lumps were excised for subsequent experiments, including immunoblotting (frozen in liquid nitrogen) and IHC (fixed in 10% formalin).

### 2.12 Statistical analysis

Data were presented as mean ± standard deviation of three or more independent experiments and analysed using GraphPad Prism 6 (GraphPad Software, San Diego, CA, USA). t test and analysis of variance were used to compare group means. Bivariate correlations were assessed using Pearson's correlation. *P* < 0.05 was considered to be statistically significant.

## 3 RESULTS

### 3.1 **METTL3 promotes the m^6^A methylation level of TFAP2C mRNA in the CDDP**‐**resistant cellular context**

**FIGURE 1:**
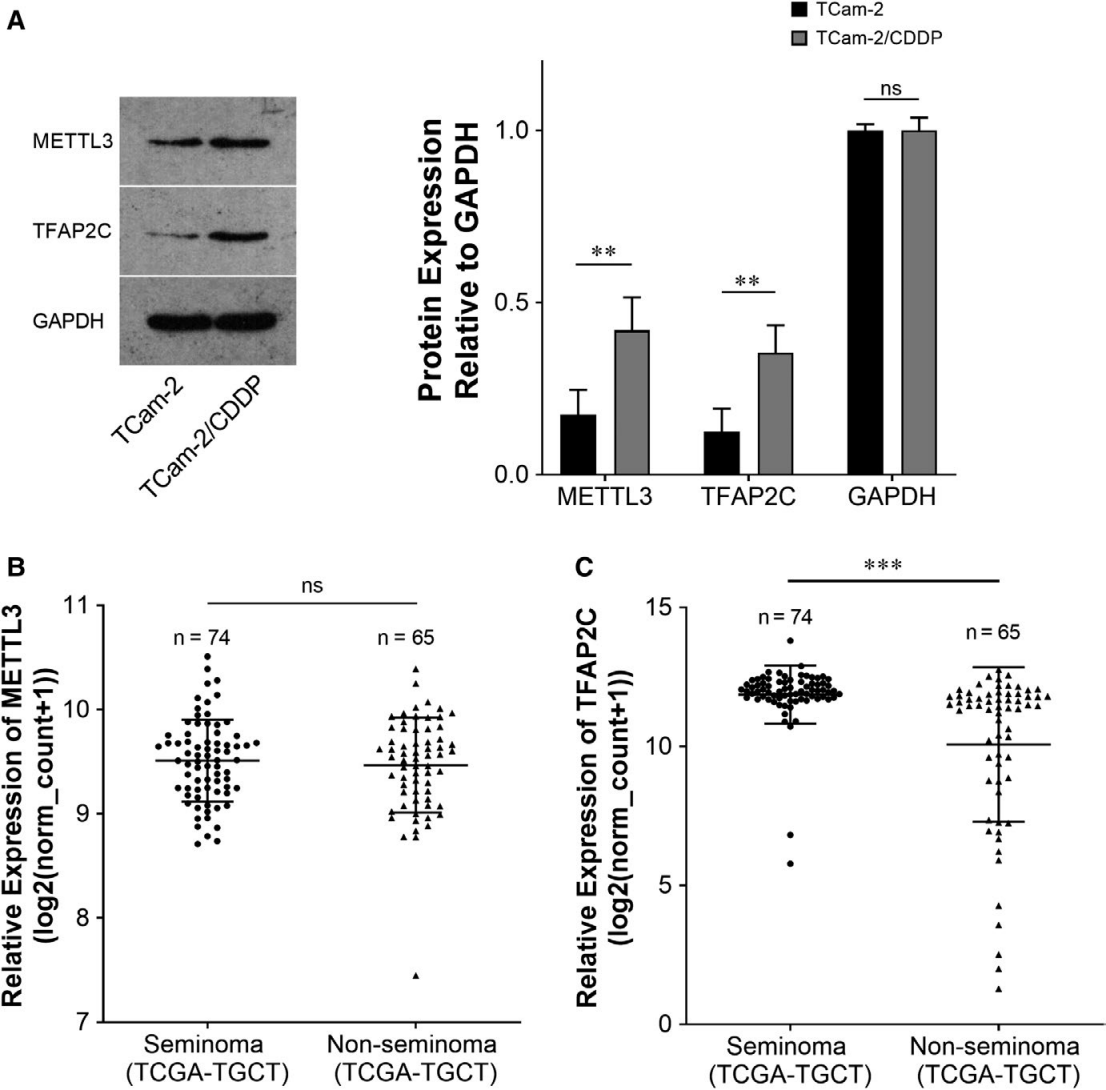
METTL3 and TFAP2C are up‐regulated in TCam‐2/CDDP cells compared to TCam‐2 cells. The expressions of METTL3 and TFAP2C protein were measured by immunoblotting. GAPDH was used as a loading control (A). Due to the difficulty in obtaining CDDP‐resistant seminoma samples, the mRNA expression levels of the aforementioned 2 genes were investigated between seminoma and non‐seminoma samples in TCGA database (B, C). CDDP, cisplatin; TCam‐2/CDPP, CDDP‐resistant TCam‐2; TCGA, The Cancer Genome Atlas. The data were presented as the mean ± SD of triplicate tests. **P < 0.01, ***P < 0.001, ns, not significant

**FIGURE 2:**
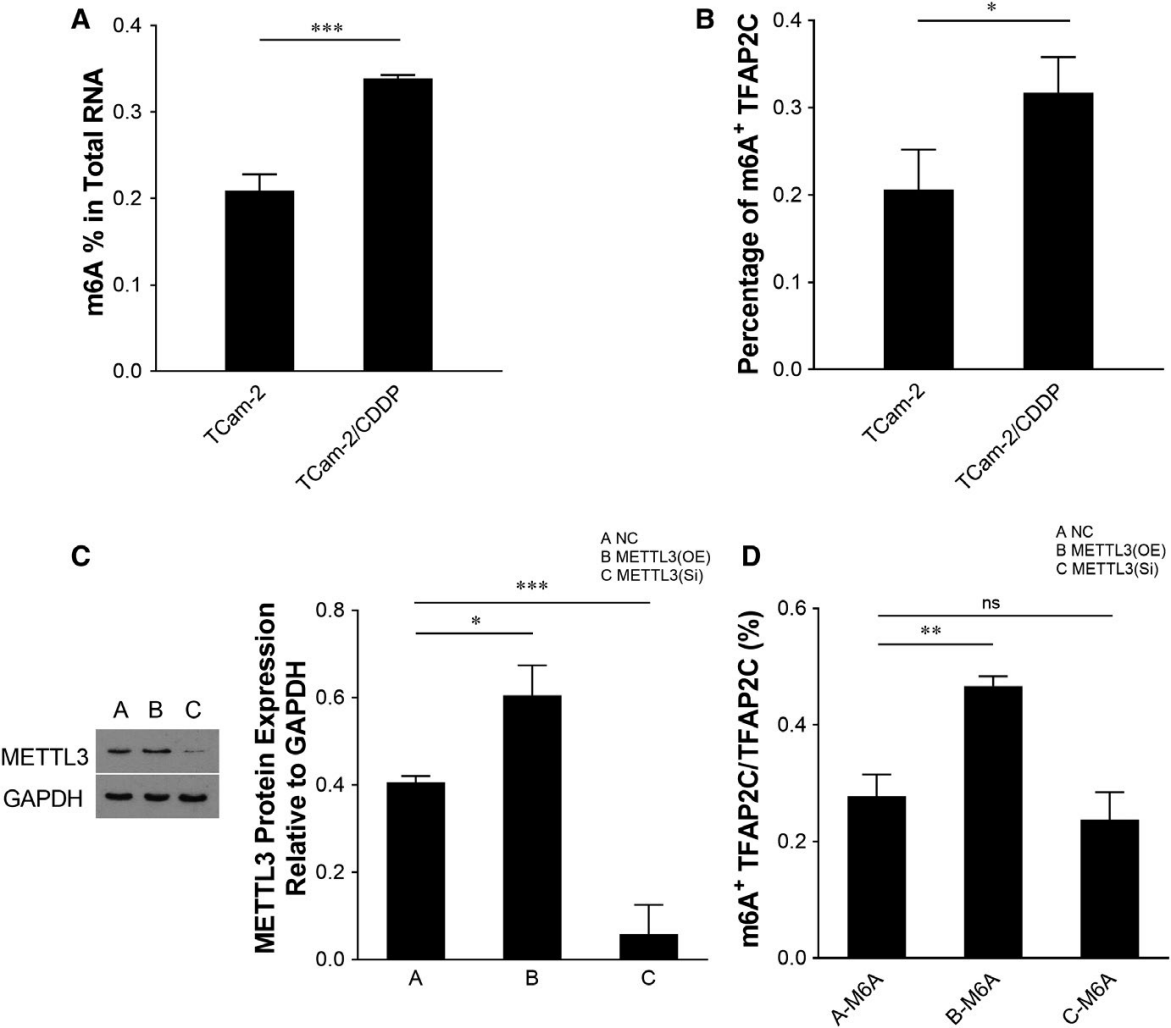
The m6A methylation levels of global cellular RNAs and TFAP2C mRNA are up‐regulated in TCam‐2/CDDP cells and TFAP2C mRNA m6A level is affected by METTL3. Total RNA m6A levels in TCam‐2 and TCam‐2/CDDP cells were measured (A). M6A immunoprecipitation combined with qPCR (M6A‐IP‐qPCR) was performed to determine the percentage of methylated TFAP2C mRNA. GAPDH was used as an internal control (B). TCam‐2/CDDP cells were transfected with empty vector control, METTL3 overexpression plasmid and METTL3 siRNA, respectively and incubated for 72 h. The expression of METTL3 protein was measured by immunoblotting. GAPDH served as a loading control (C). M6A‐IP‐qPCR was performed to determine the percentage of methylated TFAP2C mRNA. GAPDH served as an internal control (D). IP, immunoprecipitation; m6A, N6‐Methyladenosine; mRNA, messenger RNA; qPCR, quantitative reverse transcription polymerase chain reaction; siRNA, small interfering RNA. The data were presented as the mean ± SD of triplicate tests. *P < 0.05, **P < 0.01, ***P < 0.001, ns, not significant; OE, overexpression; Si, small interference

METTL3 was reported to be an m^6^A methylation ‘writer’.^32^ Our previous microarray data showed that both METTL3 and TFAP2C were up‐regulated in TCam‐2/CDDP cells compared to TCam‐2 cells.^15^ To further confirm their expressions in cell lines, immunoblotting assays were performed and protein levels of both METTL3 and TFAP2C were higher in TCam‐2/CDDP cells in comparison with TCam‐2 cells (Figure 1A). Because of the difficulty in obtaining CDDP‐resistant seminoma samples, we failed to validate their expressions in clinical samples. To partially compensate for this deficiency, we leveraged The Cancer Genome Atlas (TCGA) database and studied the mRNA levels of METTL3 and TFAP2C in seminoma and non‐seminoma samples (Figure 1B,C). For TFAP2C, the mRNA level was significantly up‐regulated in seminoma compared to non‐seminoma samples and this at least partly suggests that TFAP2C is associated with seminoma to some extent. In fact, the relations between TFAPC2 and seminoma were previously reported.^33^, ^34^, ^35^, ^36^ TFAP2C was reported to be associated with chemosensitivity.^33^ Based on detailed literature review and previous findings, we speculated that TFAP2C might be m^6^A methylated in TCam‐2 cells in the CDDP‐resistant context. The m^6^A methylation levels of global cellular RNAs and TFAP2C mRNA (Figure 2A,B) were examined, and both were up‐regulated in TCam‐2/CDDP cells, indicating that TFAP2C mRNA might be m^6^A methylated by some ‘writer’ and may contribute to cellular resistance to CDDP in TCam‐2 cells. Nettersheim et al reported that m^6^A levels in in vitro differentiated TCam‐2 were significantly increased compared to the parental TCam‐2 cells, suggesting a role of m^6^A in cell fate decisions.^28^ It is interesting that m^6^A levels are higher in both differentiated and CDDP‐resistant TCam‐2 cells compared to the parental TCam‐2 cells.

To test whether METTL3 is the right ‘writer’ of TFAP2C since METTL3 is also up‐regulated in TCam‐2/CDDP cells, METTL3 was either overexpressed or inhibited (Figure 2C) and it was found that overexpression of METTL3 increased the m^6^A level of TFAP2C mRNA (Figure 2D). These data indicate that METTL3 promotes the m^6^A methylation level of TFAP2C mRNA in TCam‐2/CDDP cells.

### 3.2 METTL3 enhances cellular viability of TCam‐2/CDDP cells under the CDDP treatment condition through increased TFAP2C mRNA stability

**FIGURE 3:**
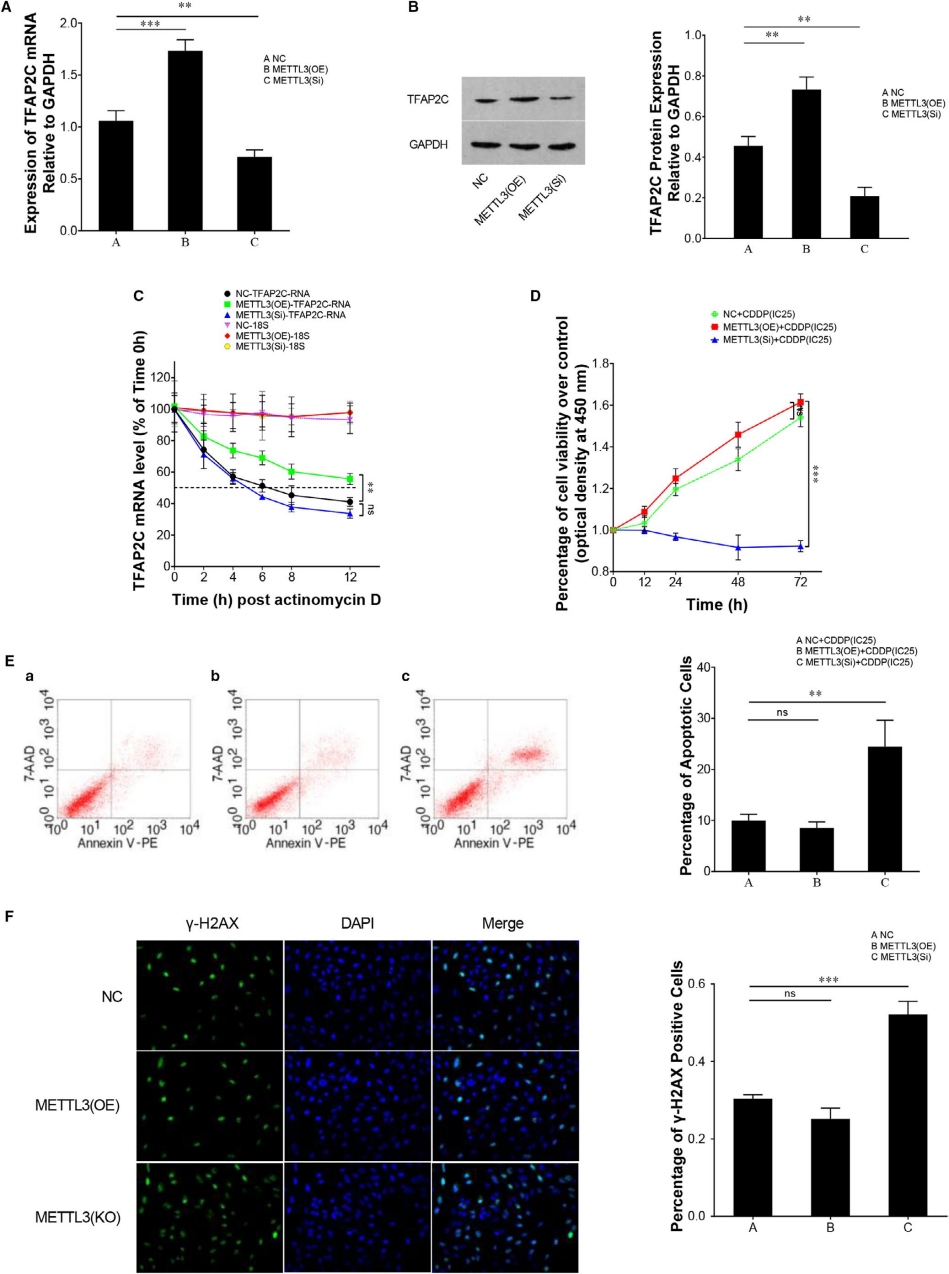
METTL3 promotes TFAP2C expression and cellular viability of TCam‐2/CDDP cells under CDDP treatment condition through regulation of TFAP2C mRNA stability. TCam‐2/CDDP cells were transfected with empty vector control, METTL3 overexpression plasmid and METTL3 siRNA, respectively, and incubated for 72 h. The expression of TFAP2C was measured by qPCR (A) and immunoblotting (B), and GAPDH was used as internal control. Another batch of transfected cells (METTL3 overexpression and interference, 72 h) was treated with actinomycin D to investigate the effect of METTL3 on decay rate of TFAP2C mRNA at different time‐points (0, 2, 4, 6, 8, 12 h). qPCR was performed to examine the relative TFAP2C mRNA level, and 18S rRNA was used as internal control (C). Dotted line represents mRNA half‐lives. To investigate the effect of METTL3 on cellular viability of TCam‐2/CDDP cells in response to CDDP treatment, the cells with METTL3 overexpression or inhibition were treated with CDDP (at the concentration of IC25) for 72 h. CCK8 cell viability assay was conducted for the determination of cell viability in cell proliferation (D). Apoptotic rate was analysed by FCM assays (E), and DNA damage rate was detected by gamma‐H2AX immunofluorescence assay (F). CCK8, Cell Counting Kit 8; DAPI, 4',6‐diamidino‐2‐phenylindole; FCM, flow cytometry; H2AX, H2A histone family member X; IC25, inhibition concentration 25%; rRNA, ribosomal RNA. The data were presented as the mean ± SD of triplicate tests. **P < 0.01, ***P < 0.001, ns, not significant

As mentioned above, METTL3 promotes m^6^A methylation of TFAP2C mRNA. m^6^A methylation of mRNA was shown to affect mRNA stability.^34^ We then further measured the mRNA and protein levels of TFAP2C after METTL3 was overexpressed or inhibited (Figure 3A). A higher level of METTL3 resulted in up‐regulation of TFAP2C, and a lower level of METTL3 resulted in down‐regulation of TFAP2C (Figure 3B). The up‐regulation of TFAP2C was speculated to be caused by the enhanced mRNA stability since m^6^A methylation was delineated as an mRNA stability mechanism. To confirm this point, actinomycin D was introduced into TCam‐2/CDDP cells and RNA decay assays revealed that METTL3 overexpression increased the mRNA stability of TFAP2C mRNA (Figure 3C). As METTL3 was up‐regulated in TCam‐2/CDDP cells, we then inhibited METTL3 using siRNAs or overexpressed METTL3 using plasmids and examined the effect of METTL3 on cellular viability of TCam‐2/CDDP cells in response to CDDP treatment. When treated with CDDP at inhibitory concentration of 25% (IC25),^15^, ^31^ METTL3 overexpressing cells showed enhanced viability and reduced apoptosis rate. On the contrary, inhibition of METTL3 led to worse survival and increased apoptosis rate in TCam‐2/CDDP cells (Figure 3D,E). Cell cycle progression is tightly correlated with DNA damage. Studies indicated that when DNA damage occurred, cells either transiently blocked cell cycle progression to spare time for DNA repair or exited the cell cycle.^35^ From this point of view, we further checked the DNA damage status of TCam‐2/CDDP cells in the CDDP treatment context after METTL3 overexpression or inhibition and DNA damage was attenuated in the METTL3‐inhibited condition (Figure 3F). These data together demonstrate that METTL3 enhances viability of TCam‐2/CDDP cells in response to CDDP.

### 3.3 IGF2BP1 functions as a TFAP2C m^6^A ‘reader’ and enhances TFAP2C mRNA stability

**FIGURE 4:**
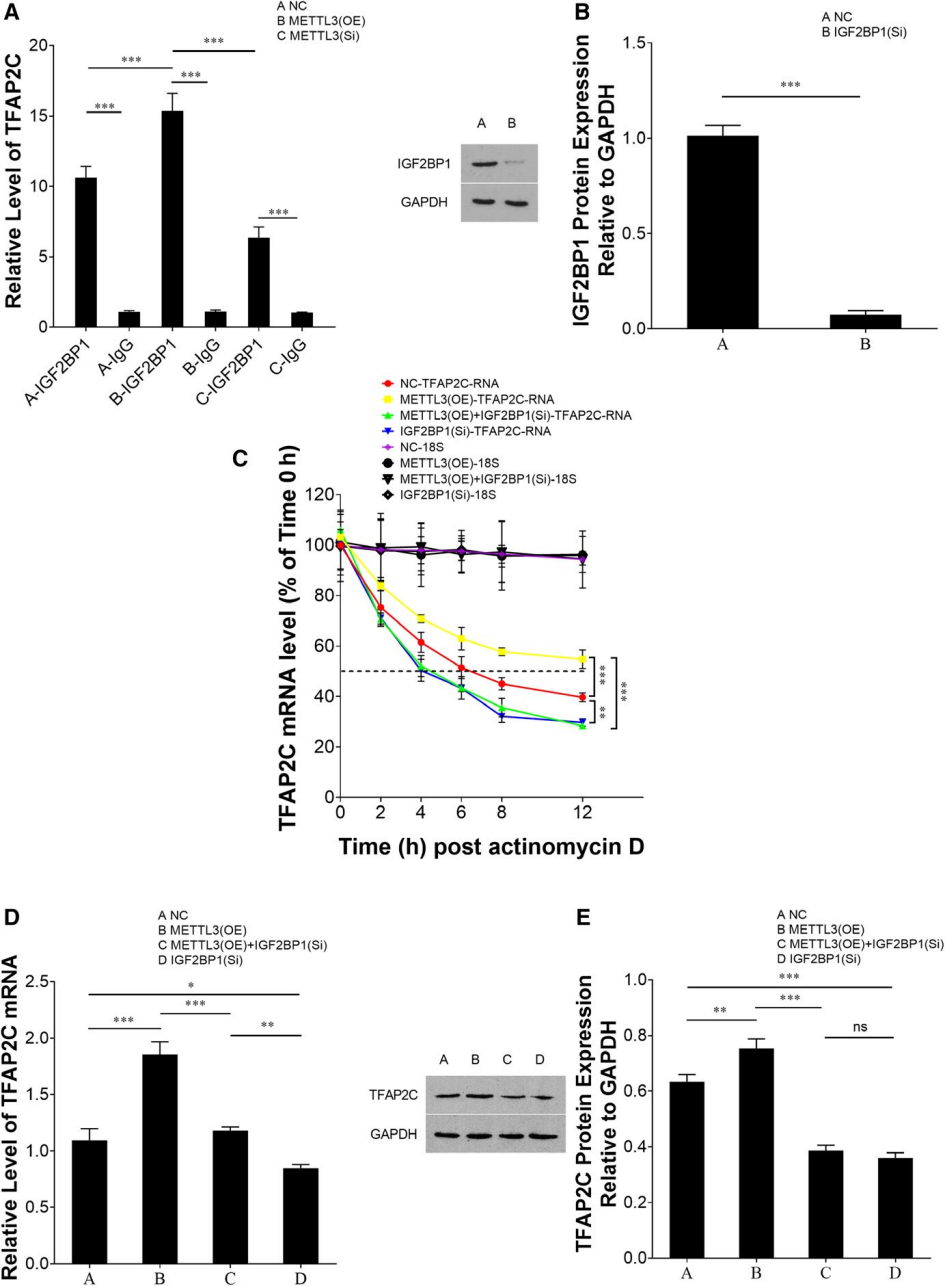
IGF2BP1 binds to TFAP2C and enhances TFAP2C mRNA stability. METTL3 was overexpressed or inhibited in TCam‐2/CDDP cells in the same way as described previously. RNA immunoprecipitation (RIP) assays were performed to determine the combination between IGF2BP1 and TFAP2C (A). In mRNA stability assay, IGF2BP1 was inhibited (transfected with IGF2BP1 siRNA) independently or together with METTL3 overexpression before introduction of actinomycin D in TCam‐2/CDDP cells, and qPCR was performed at several time‐points (0, 2, 4, 6, 8, 12 h) to examine the relative mRNA level, and 18S rRNA was used as internal control (B, C). Dotted line represents mRNA half‐lives. Besides, qPCR (D) and immunoblotting assays (E) were conducted to study the overall and individual effect of IGF2BP1 inhibition and METTL3 overexpression on TFAP2C expression. The data were presented as the mean ± SD of triplicate tests. *P < 0.05, **P < 0.01, ***P < 0.001, ns, not significant

It is well established that IGF2BP1 acts as an m^6^A ‘reader’.^36^ In our study, we conducted exploratory research to annotate the possible role of IGF2BP1 in cellular resistance to CDDP. RNA immunoprecipitation (RIP) assays were performed using either IGF2BP1 antibody or IgG antibody (negative control). Compared to the IgG group, TFAP2C was significantly enriched by the IGF2BP1 antibody, which indicated that IGF2BP1 bound to TFAP2C (Figure 4A). Furthermore, when METTL3 was overexpressed, which meant that the m^6^A level of TFAP2C was increased (as confirmed previously), more TFAP2C was enriched by the IGF2BP1 antibody compared to negative control. Inhibition of METTL3 resulted in less enrichment of TFAP2C (Figure 4A). This partly indicated that IGF2BP1 recognized the m^6^A site of TFAP2C mRNA. The effect of IGF2BP1 on TFAP2C mRNA stability was explored leveraging actinomycin D. As described previously, overexpression of METTL3 promoted TFAP2C mRNA stability. However, when IGF2BP1 siRNA was introduced into METTL3‐overexpressing cells, a higher mRNA decay rate was obtained (Figure 4B,C). In addition, the mRNA and protein levels of TFAP2C were measured to study the overall and individual effects of IGF2BP1 inhibition and METTL3 overexpression on TFAP2C expression. The up‐regulation of TFAP2C expression caused by METTL3 overexpression was reversed by IGF2BP1 inhibition (Figure 4D,E). Thus, IGF2BP1 is putative to be a TFAP2C m^6^A ‘reader’ and it enhances TFAP2C mRNA stability.

### 3.4 Inhibition of IGF2BP1 and TFAP2C attenuates cellular viability of TCam‐2/CDDP cells in response to CDDP

**FIGURE 5:**
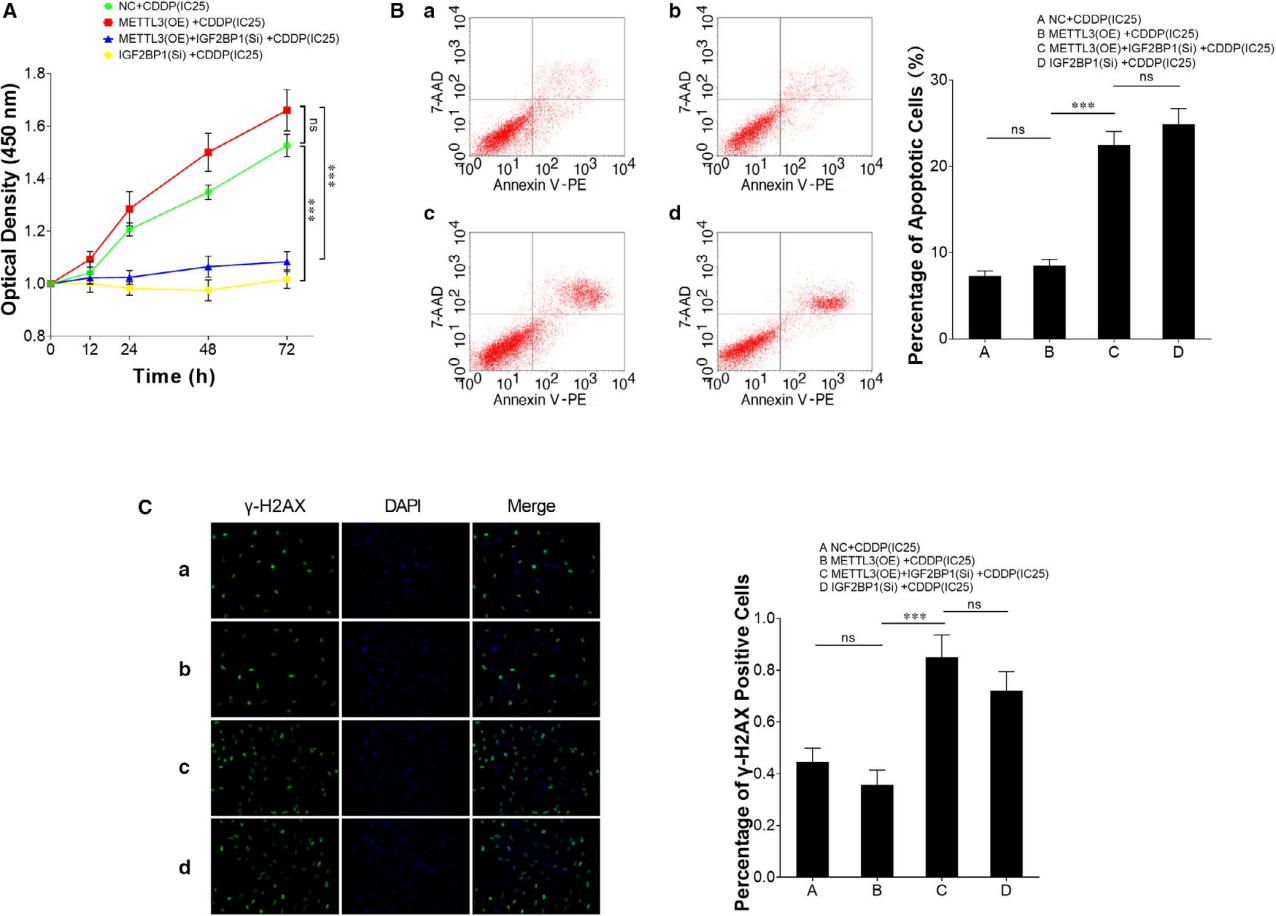
Inhibition of IGF2BP1 attenuates cellular viability of TCam‐2/CDDP cells under CDDP treatment condition. IGF2BP1 was inhibited (IGF2BP1 siRNA) independently or together with METTL3 overexpression in TCam‐2/CDDP cells and cells were treated with CDDP (at the concentration of IC25) for 72 h. CCK8 cell viability assay was performed to determine cell viability (A). Apoptotic rate (B) was analysed by FCM assays. DNA damage rate was detected by gamma‐H2AX immunofluorescence assay (C). The data were presented as the mean ± SD of triplicate tests. *P < 0.05, ***P < 0.001, ns, not significant

**FIGURE 6:**
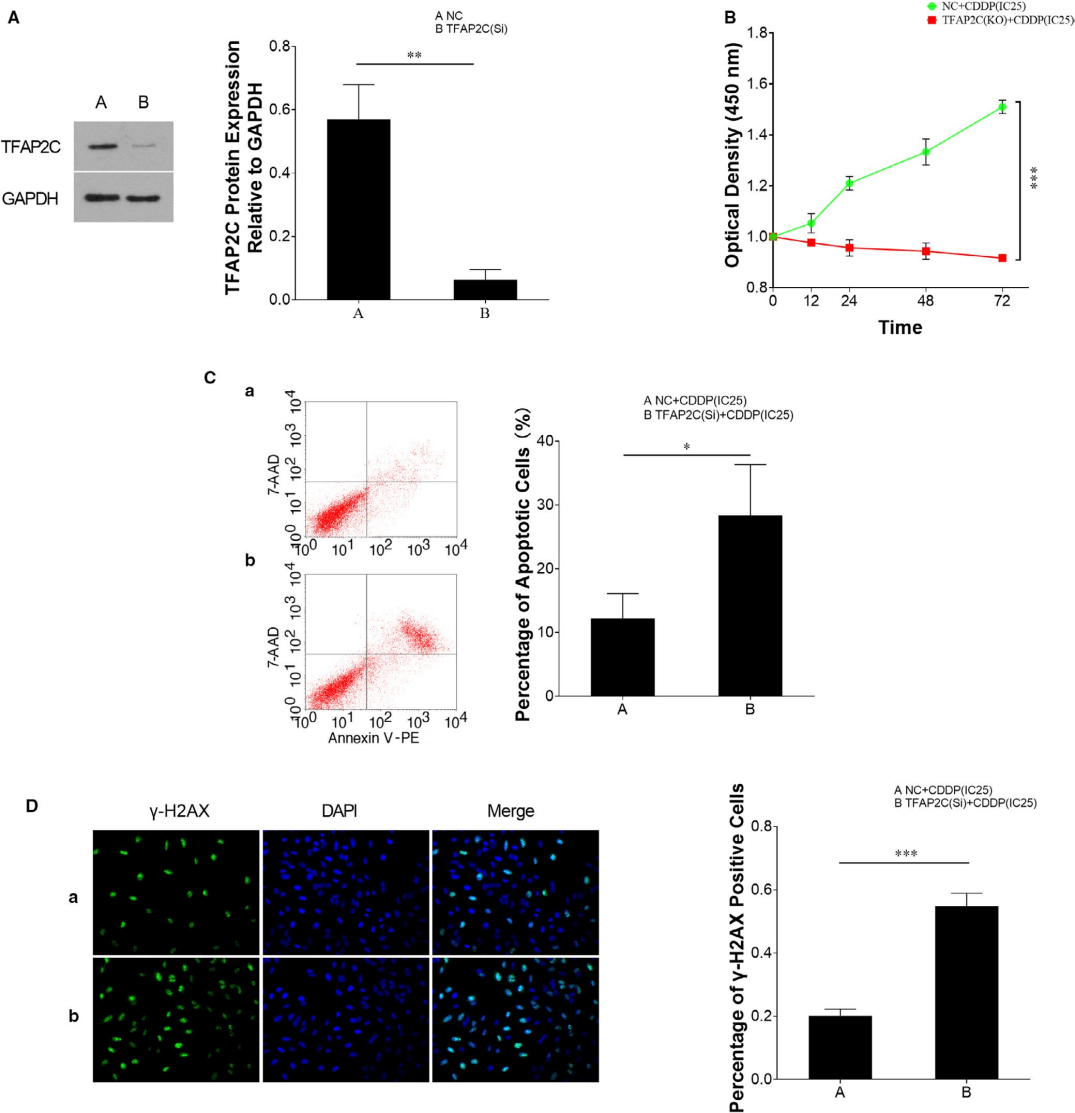
Inhibition of TFAP2C attenuates cellular viability of TCam‐2/CDDP cells under CDDP treatment condition. TCam‐2/CDDP cells were transfected with TFAP2C siRNAs (A) and treated with CDDP (at the concentration of IC25) for 72 h. Likewise, CCK8 cell viability assay was performed to determine cell viability (B). Apoptotic rate (C) was analysed by FCM assays. DNA damage rate was detected by gamma‐H2AX immunofluorescence assay (D). The data were presented as the mean ± SD of triplicate tests. *P < 0.05, **P < 0.01, ***P < 0.001

Taking the above‐mentioned findings into account, IGF2BP1 was hypothesized to drive cellular resistance to CDDP treatment in TCam‐2/CDDP cells. To validate this hypothesis, IGF2BP1 was inhibited (IGF2BP1 siRNA) independently or together with METTL3 overexpression in TCam‐2/CDDP cells and cells were treated with CDDP. CCK8 cell viability assay showed that enhanced cellular viability caused by METTL3 overexpression was reversed by IGF2BP1 inhibition (Figure 5A). A higher apoptosis rate was also observed in IGF2BP1‐inhibited cells compared to cells without IGF2BP1 inhibition (irrespective of whether with or without METTL3 overexpression) (Figure 5B). DNA damage status was also detected, and inhibition of IGF2BP1 led to an increased DNA damage rate (Figure 5C). Similarly, we inhibited TFAP2C in TCam‐2/CDDP cells using siRNAs (Figure 6A) and performed these assays. Inhibition of TFAP2C resulted in attenuated viability and more apoptotic cells (Figure 6B,C). More DNA damage was detected after TFAP2C inhibition (Figure 6D). These data provide strong proof that IGF2BP1 and TFAP2C play crucial roles in enhancing cellular survival in CDDP treatment stress.

### 3.5 TFAP2C activates DNA repair genes WEE1 G2 checkpoint kinase (WEE1) and breast cancer type 1 (BRCA1)

**FIGURE 7:**
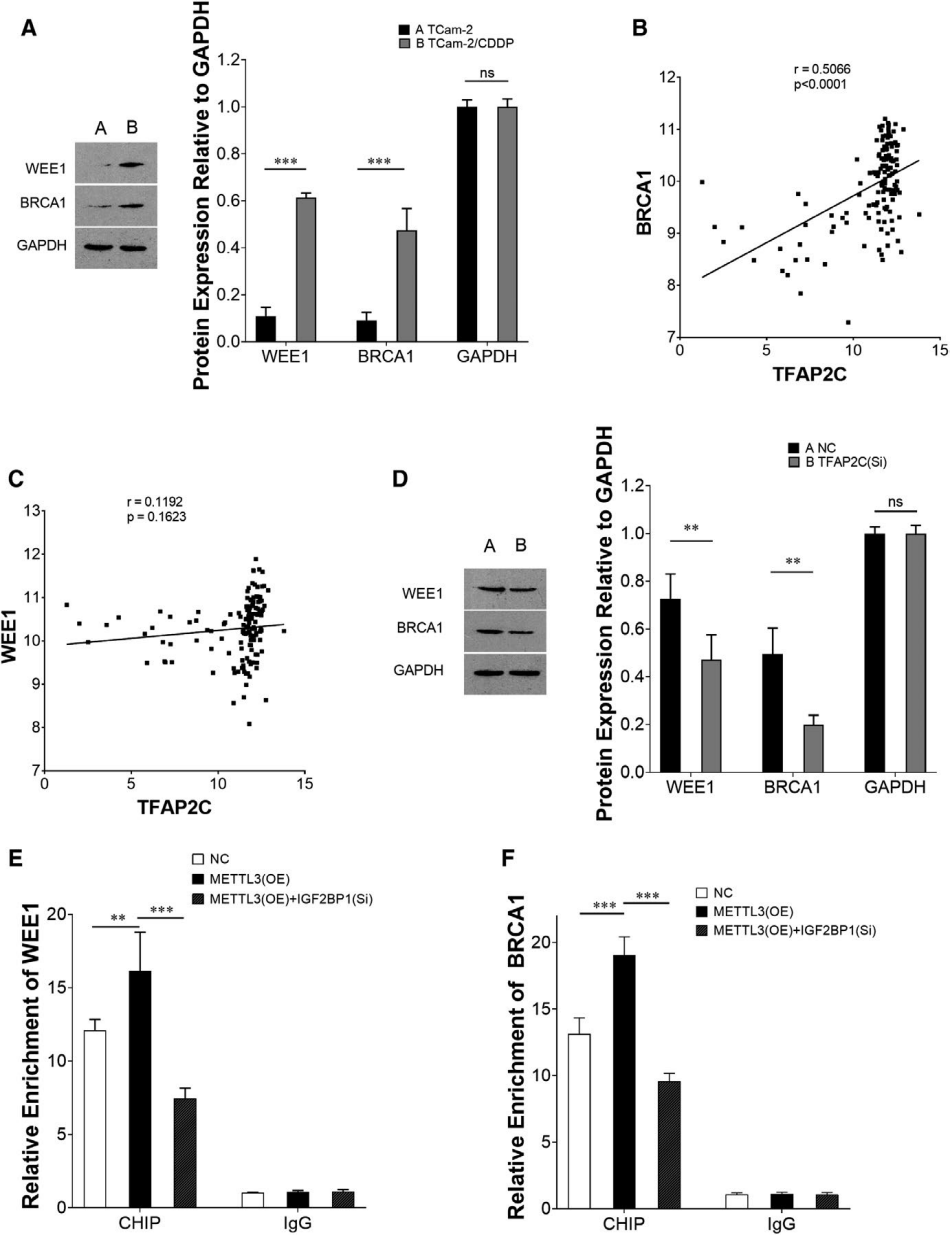
DNA repair gene WEE1 and BRCA1 were activated by TFAP2C. The protein expression levels of WEE1 and BRCA1 were investigated between TCam‐2 and TCam‐2/CDDP cells (A). The correlation of their mRNA levels with TFAP2C was analysed (Pearson's Correlation) using TCGA database (B, C). To further determine their relationships, TFAP2C was inhibited (TFAP2C siRNA) in TCam‐2/CDDP cells and immunoblotting assays were performed to measure the protein levels of WEE1 and BRCA1 (D). Chromatin immunoprecipitation (ChIP) assays were performed to measure the enrichment of WEE1 (E) and BRCA1 (F) after METTL3 was overexpressed independently or together with IGF2BP1 inhibition, which changed the mRNA stability status of TFAP2C. The data were presented as the mean ± SD of triplicate tests. *P < 0.05, **P < 0.01, ***P < 0.001, ns, not significant

As described above, more DNA damage was related to decreased cell viability in TCam‐2/CDDP cells. DNA repair mechanism plays important roles in cellular drug resistance. Since TFAP2C promoted cellular viability and was found to be negatively correlated with DNA damage, we further investigated whether TFAP2C was a member in DNA repair pathways. We reviewed our microarray data and screened DNA repair genes WEE1 and BRCA1 due to their higher expression in TCam‐2/CDDP cells compared to TCam‐2 cells. Higher expressions of WEE1 and BRCA1 in TCam‐2/CDDP cells compared to TCam‐2 cells were confirmed using immunoblotting (Figure 7A). Both WEE1 and BRCA1 showed higher expression in seminoma compared to non‐seminoma samples according to the TCGA database (Figure S1), and the expression of BRCA1 was significantly correlated with TFAP2C (Figure 7B). However, the expression of WEE1 was significantly correlated with TFAP2C (Figure 7C). When TFAP2C was inhibited in TCam‐2/CDDP cells, immunoblotting assays showed decreased protein levels of WEE1 and BRCA1 (Figure 7D). ChIP assays were further performed to measure the enrichment of WEE1 and BRCA1 using the TFAP2C antibody compared to the IgG antibody. Both WEE1 and BRCA1 were significantly enriched by TFAP2C. Besides, overexpressed METTL3 promoted the enrichment, which was reversed by IGF2BP1 inhibition (Figure 7E,F). By combining the previous results, we showed that m^6^A methylated‐TFAP2C activated DNA repair genes WEE1 and BRCA1 and affected the cellular response to CDDP treatment stress.

### 3.6 In vivo effect of the METTL3/IGF2BP1/TFAP2C signalling pathway on CDDP sensitivity in seminoma

**FIGURE 8:**
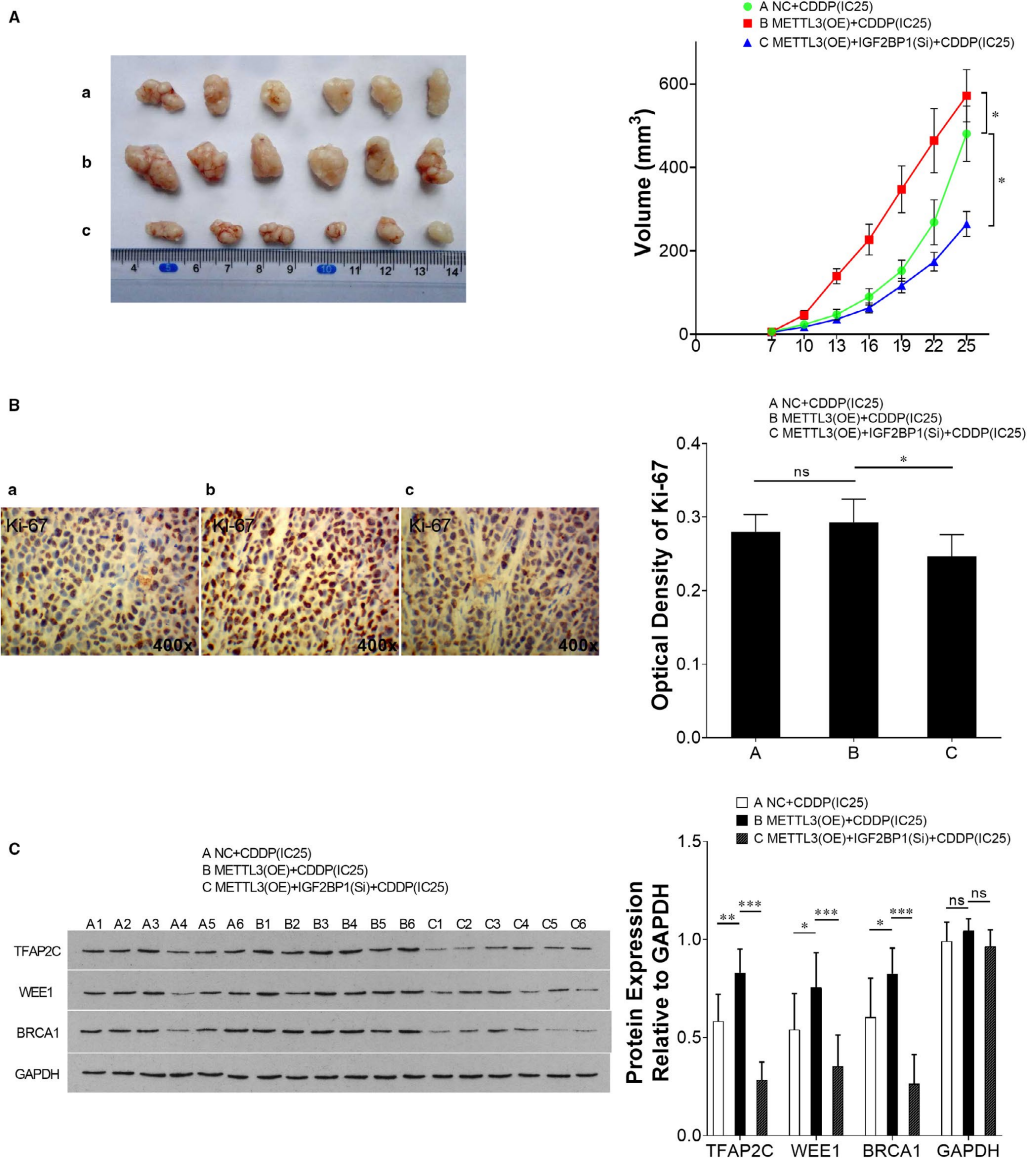
The effect of METTL3/IGF2BP1/TFAP2C on CDDP sensitivity of seminoma in vivo. Male mice were subcutaneously injected with tumour cells to establish xenografts (METTL3‐overexpressed TCam‐2/CDDP cells were inoculated at the initial time for once and IGF2BP1‐inhibited TCam‐2/CDDP cells were inoculated every 3 days). And at the same time, those mice were intraperitoneally injected with CDDP (6 mg/kg bodyweight) or vehicle every week for three doses. Then, the mice were humanely killed and their tumours were excised 25 days after inoculation. Tumour volumes were measured every third day for consecutive seven times (A). Representative photographs of IHC staining of Ki‐67 are presented (B). The protein expression levels of TFAP2C, WEE1 and BRCA1 were analysed by immunoblotting. GAPDH served as loading control (C). The data were presented as the mean ± SD of triplicate tests. IHC, immunohistochemistry. *P < 0.05, **P < 0.01, ***P < 0.001, ns, not significant

Male mice were subcutaneously injected with tumour cells and treated with CDDP. Consistent with in vitro experiments, in vivo tumours derived from TCam‐2/CDDP cells with METTL3 overexpression grew more rapidly than negative control under CDDP treatment and the growth was inhibited by IGF2BP1 inhibition (Figure 8A). This inhibitory effect on growth induced by IGF2BP1 inhibition was also confirmed by IHC staining of the proliferation marker Ki‐67 in xenografts (Figure 8B). Compared to negative control, METTL3 overexpression did not significantly enhance Ki‐67 staining, which might be due to the limited quantity of xenografts (Figure 8B). We also performed immunoblotting to measure the expression of proteins. Similar to in vitro assays, METTL3 overexpression resulted in up‐regulation of TFAP2C, WEE1 and BRCA1 and this effect was reversed by IGF2BP1 inhibition (Figure 8C). Collectively, we showed the interactions of the METTL3/IGF2BP1/TFAP2C signalling pathway and unveiled its contributions to in vivo CDDP resistance in seminoma.

## 4 DISCUSSION

The chemotherapeutic efficacy of CDDP is significantly hampered by the onset of chemoresistance. Compared to non‐seminomas of type II TGCTs, seminoma is more curable. However, seminoma is also lethal when gaining resistance to therapy. Currently, there is no tailored therapy for cisplatin‐resistant seminoma and the outcome of relapsed cases is dismal. CDDP resistance in seminoma is a topic that has been scarcely investigated. Epigenetic modifications, such as RNA methylation and miRNA regulation, may play important roles in tumoral resistance to chemotherapy.^15^ This study unveils a new participator, m^6^A modification in chemoresistance of seminoma. In the past few years, m^6^A has been found to be associated with numerous disease development processes, including chemoresistance.^37^, ^38^, ^39^ Proteins involved in m^6^A methylation mainly include m^6^A methyltransferase, demethyltransferase and recognizer, which are termed as ‘writer’, ‘eraser’ and ‘reader’, respectively. Their interactions exquisitely regulate m^6^A methylation.^40^ In the present study, enhanced stability of TFAP2C mRNA affected CDDP efficacy in seminoma TCam‐2 cells. The stability of TFAP2C mRNA was regulated through m^6^A modification, in which METTL3 acted as a ‘writer’ and IGF2BP1 as a ‘reader’. The m^6^A methylation on TFAP2C subsequently led to overexpression of DNA repair‐related genes WEE1 and BRCA1 and conferred cellular resistance to CDDP treatment.

METTL3 is one of the most well‐known writer components of m^6^A.^41^ As indicated by its name, METTL3 contributes to m^6^A installation on RNAs and this function has been widely confirmed. For instance, knockdown of METTL3 reduces m^6^A enrichment in mouse embryonic stem cells and HepG2 cells.^24^, ^42^, ^43^ METTL3 is known to promote translation in human tumour cells.^44^, ^45^ Yue et al showed that METTL3‐mediated m^6^A methylation is a crucial factor for epithelial‐mesenchymal transition and gastric cancer metastasis.^46^ Reduced METTL3 is delineated to down‐regulate the m^6^A level and increase the apoptosis rate in Hela cells.^47^ Our data showed that METTL3 promoted m^6^A of TFAP2C and attenuation of METTL3 resulted in elevated apoptosis. Furthermore, METTL3 promotes chemoresistance in pancreatic cancer cells.^48^ We also found a similar effect of METTL3 in seminoma. It is worth noting that this does not necessarily mean that METTL3 directly promotes chemoresistance. As a ‘writer’ in m^6^A modification, METTL3 interacts with its targets and the biological significance largely relies on the role of the targets.

In this study, the ‘reader’ IGF2BP1 is a conserved RNA‐binding protein that is proven to act as a key regulator of mRNA metabolism, including mRNA stabilization and translation.^49^, ^50^, ^51^ IGF2BP1 participates in the transportation of specific mRNAs, and its function depends on impediment of mRNA decay.^52^, ^53^ IGF2BP1 hinders the degradation of target transcripts, in a way potentially opposite to miRNA‐directed decay.^54^ Studies have revealed that m^6^A enhances the interaction of IGF2BP1 with its target mRNAs, suggesting the m^6^A‐reader role of IGF2BP1.^55^ Expression of IGF2BP1 has been reported in a panel of human tumours, and substantial data have shown that IGF2BP1 drives tumorigenesis both in vitro and in vivo.^54^, ^56^ Though IGF2BP1 is annotated as a tumour driver, its role in promoting chemoresistance of tumour cells remains poorly understood.^56^ In our assays in the present study, the biological effect of METTL3 overexpression on seminoma cells was attenuated or reversed by inhibition of IGF2BP1, suggesting a close relation and synergism of the ‘writer’ and ‘reader’ in m^6^A modification.

TFAP2C belongs to a conserved family of DNA‐binding transcription factors.^56^ TFAP2C is scarcely detectable in normal tissues, and it reappears in a panel of tumours.^57^, ^58^, ^59^ Besides, high expression of TFAP2C is correlated with poor survival in certain types of tumours.^33^, ^60^ The significance of TFAP2C in anti‐tumour treatment is depicted in several studies. In invasive breast cancer, TFAP2C contributes to poor response to anti‐hormone therapy^61^; and in colorectal cancer, it enhances chemotherapeutic resistance.^33^ As the m^6^A target of METTL3 found in this study, TFAP2C was found to potentiate chemoresistance, which is consistent with the findings in colorectal cancer. In addition, we also revealed that TFAP2C activates certain DNA repair genes.

Genomic instability is a hallmark of tumours and is associated with accumulated DNA damage. Tumour cells are known to activate specific processes in response to DNA damage, which are termed DNA damage response.^62^, ^63^ The activated processes either cause DNA repair, which promotes survival, or activate an apoptotic response.^64^ The cytotoxic effects of CDDP rely on DNA repair mechanisms to a great extent since it is a DNA alkylating agent.^65^, ^66^ Emerging evidence shows that potentiated DNA repair contributes greatly to cisplatin resistance.^67^ Therefore, DNA repair is a vital target to improve tumour therapy, and therapeutic strategy aiming to inhibit a DNA damage response is set forth as a promising approach against drug resistance since it might re‐sensitize tumour cells to chemotherapeutic agents.^68^ WEE1 and BRCA1 are well‐known participators in a DNA damage response.^69^, ^70^, ^71^ Inhibition of WEE1 was shown to potentiate the efficiency of chemotherapeutic agents.^68^, ^72^ In this study, both WEE1 and BRCA1 were found to be up‐regulated by enhanced TFAP2C mRNA stability and they drove tumour growth in vivo.

Although m^6^A is one of the most prevalent manners among numerous types of RNA modifications identified, its biological significance in TGCTs awaits further proof.^37^ Our work might improve our understanding of m^6^A modification in refractory seminoma. Besides, in consideration of the dual roles of m^6^A in tumorigenesis, which could either confer tumour progression or tumour suppression, it is interesting to know whether m^6^A promotes chemoresistance or in a similar manner plays dual roles in cellular response to chemotherapy.^19^ At least based on our findings, m^6^A of TFAP2C promotes TCam‐2 cell survival and confers resistance to CDDP in seminoma.

However, some limitations of this study should be acknowledged. Owing to the difficulty in obtaining samples from CDDP‐resistant seminoma cases, we were not able to detect the expressions of METTL3 and TFAP2C in clinical chemo‐resistant samples. How TFAP2C regulates WEE1 and BRCA1 remains unknown, and it warrants further exploration.

Collectively, this study updates our knowledge of the mechanisms underlying chemoresistance in seminoma. In seminoma, m^6^A methylation of TFAP2C mRNA initiated by METTL3, in conjunction with the other mechanisms, gives the tumour cells more time to develop resistance to CDDP treatment. Hopefully, these findings will provide new insights into strategies for circumventing cisplatin resistance and identifying promising targets for potentiating chemotherapeutic efficacy in patients with seminoma.

## CONFLICT OF INTEREST

The authors have no conflict of interest.

## AUTHOR CONTRIBUTION

**Jingchao Wei:** Conceptualization (lead); data curation (lead); formal analysis (lead); funding acquisition (supporting); investigation (lead); methodology (lead); project administration (lead); resources (lead); software (lead); supervision (lead); validation (lead); visualization (lead); writing – original draft (lead); writing – review and editing (lead). **Yinghao Yin:** Conceptualization (equal); data curation (equal); investigation (equal); methodology (equal); resources (equal); validation (equal); visualization (equal); writing – original draft (equal). **Jun Zhou:** Data curation (supporting); investigation (supporting); methodology (supporting). **Hanfei Chen:** Investigation (supporting); validation (supporting); visualization (supporting). **Jingxuan Peng:** Formal analysis (supporting); methodology (supporting); software (supporting). **Jianfu Yang:** Conceptualization (supporting); funding acquisition (supporting); project administration (supporting); supervision (supporting); writing – review and editing (supporting). **Yuxin Tang:** Conceptualization (lead); funding acquisition (lead); investigation (lead); project administration (lead); resources (lead); supervision (lead); validation (lead); writing – review and editing (lead).

## Supporting information

Fig S1

## Data Availability

The data generated and analysed during this study are available upon reasonable request from the corresponding author.
