# Supplementary figures and images for: METTL3 potentiates resistance to cisplatin through m6A modification of TFAP2C in seminoma
Source: J Cell Mol Med. 2020 Aug 28;24(19):11366–80. doi: 10.1111/jcmm.15738 (PMC7576266; doi:10.1111/jcmm.15738)

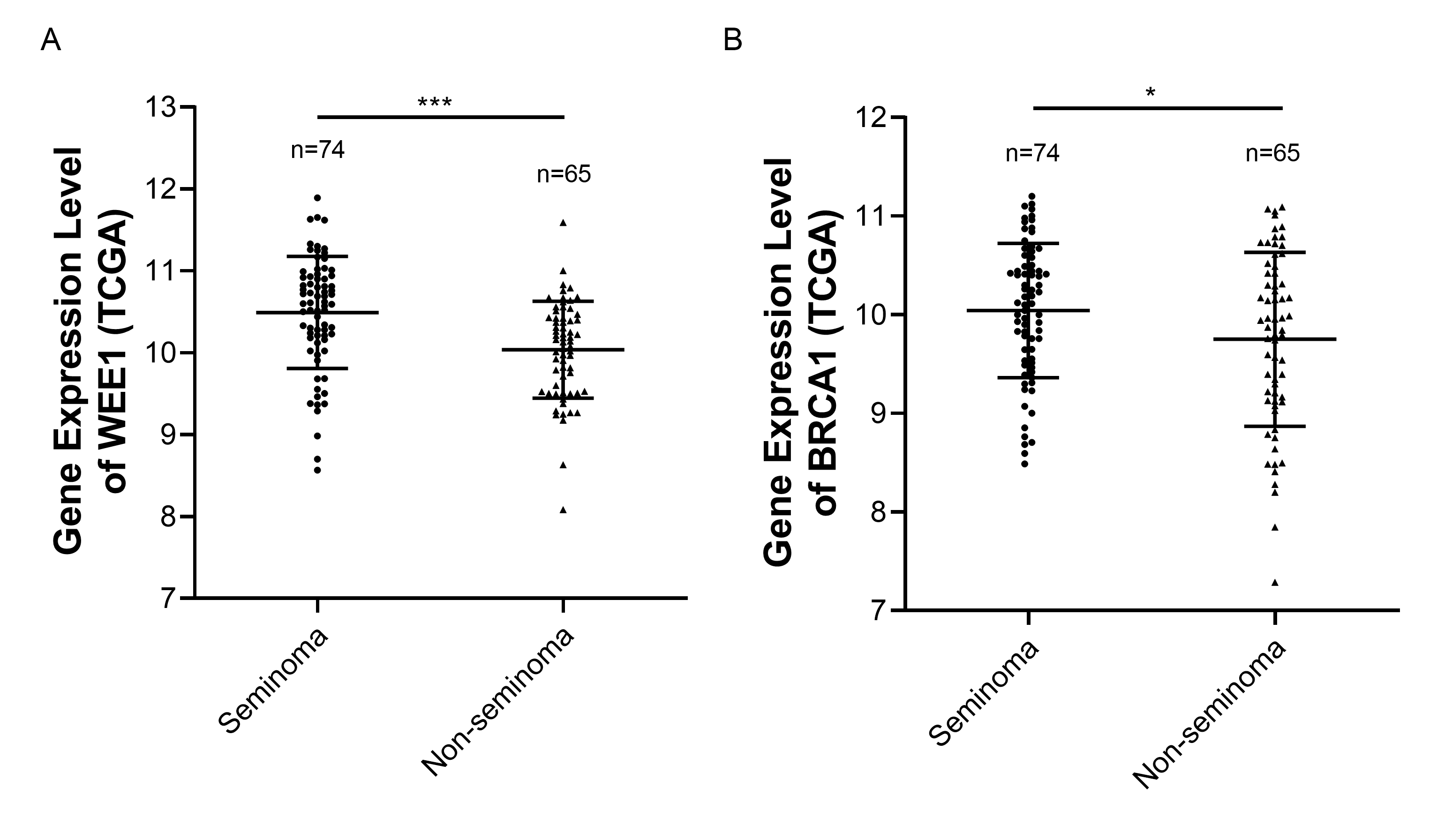

Supplement: Supplementary file 1 — Fig S1 [file JCMM-24-11366-s001.tif]
